# Cancer immunotherapy by targeting immune checkpoints: mechanism of T cell dysfunction in cancer immunity and new therapeutic targets

**DOI:** 10.1186/s12929-017-0341-0

**Published:** 2017-05-25

**Authors:** Hwei-Fang Tsai, Ping-Ning Hsu

**Affiliations:** 1 Department of Internal Medicine, Taipei Medical University Shuang Ho Hospital, New Taipei City, Taiwan; 2 Gradute Institute of Clinical Medicine, Taipei Medical University, Taipei, Taiwan; 3 Graduate Institute of Immunology, College of Medicine, National Taiwan University, No. 1, Sec. 1, Jen-Ai Rd, Taipei, 100 Taiwan; 4 Department of Internal Medicine, National Taiwan University Hospital, Taipei, Taiwan

**Keywords:** Cancer immunotherapy, Immune checkpoint, T cell exhaustion, New therapeutic targets

## Abstract

Immune checkpoints or coinhibitory receptors, such as cytotoxic T lymphocyte antigen (CTLA)-4 and programmed death (PD)-1, play important roles in regulating T cell responses, and they were proven to be effective targets in treating cancer. In chronic viral infections and cancer, T cells are chronically exposed to persistent antigen stimulation. This is often associated with deterioration of T cell function with constitutive activation of immune checkpoints, a state called ‘exhaustion’, which is commonly associated with inefficient control of tumors and persistent viral infections. Immune checkpoint blockade can reinvigorate dysfunctional/exhausted T cells by restoring immunity to eliminate cancer or virus-infected cells. These immune checkpoint blocking antibodies have moved immunotherapy into a new era, and they represent paradigm-shifting therapeutic strategies for cancer treatment. A clearer understanding of the regulatory roles of these receptors and elucidation of the mechanisms of T cell dysfunction will provide more insights for rational design and development of cancer therapies that target immune checkpoints. This article reviews recent advance(s) in molecular understanding of T cell dysfunction in tumor microenvironments. In addition, we also discuss new immune checkpoint targets in cancer therapy.

## Background

Cancer evades antitumor immune attacks via both inhibiting recognition of cancer specific antigens by T cells and causing dysfunction of CD8 cytotoxic T cells (CTL). Recent breakthroughs and encouraging clinical results with various immune checkpoint inhibitors, such as anti-PD-1 monoclonal antibodies (mAbs) and anti-CTLA-4 mAbs, have demonstrated tremendous potential to control cancer by immune activation [1–9]. Immune checkpoint blockade is able to reinvigorate dysfunctional/exhausted T cells by restoring tumor-specific immunity to eliminate cancer cells. In addition to melanoma, inspiring results were reported in other cancers including lung cancer, renal cell carcinoma, bladder cancer, and additional approvals are expected, indicating the great promise held by these mAbs. All these results clearly indicate that a new era of immunotherapy has arrived. Long-term control of cancer with durable treatment response now seems achievable. These mAbs have added a new cornerstone to immunotherapy, making it,another key pillar for cancer treatment in the near future. Immune checkpoint blockade has greatly expanded our knowledge of antitumor immunity and has introduced radical changes and new trends in cancer therapy. Moreover, multiple new immune checkpoints that represent potential new targets for cancer therapy are now under active development. This article reviews advance(s) in recent molecular understanding of T cell dysfunction within tumor microenvironments and of developments of new immune checkpoint therapeutic targets for cancer.

### Immune checkpoints or coinhibitory receptors play critical roles in immune homeostasis

To eradicate tumor cells and induce antitumor immunity, T cells are able to recognize tumor antigens presented to T cell receptors (TCRs) by antigen-presenting cells (APCs). After binding to TCR, a second signal (signal two, also called costimulatory signal) is needed for T cell activation. The costimulatory signal comes from the binding of CD28 molecule on T cells with its ligand, B-7 molecules (CD80 and CD86) on APCs. CTLA-4, an immune checkpoint or coinhibitory receptor is induced after T cell activation. CTLA-4 has a higher binding affinity for B-7 ligands than CD28, and CTLA-4 can bind to B7 and displace CD28, leading to attenuation and termination of T cell responses and establishment of tolerance, to minimize the development of autoimmunity. Immune checkpoints or coinhibitory receptors have a central role in regulating autoimmunity, and deficiency of CTLA-4 develops profound lymphoproliferation and systemic autoimmune disease [10, 11]. PD-1 pathway was recognized to play a regulatory role in inhibiting T cell activation and restraining T cell function [12, 13], and PD-1 knockout mice developed proliferative arthritis and a lupus-like autoimmune diseases [14]. Many checkpoint receptors have been genetically associated with autoimmunity and inflammatory diseases [15–18], suggesting that immune checkpoints or coinhibitory receptors play a critical role in immune tolerance and regulating homeostasis. Therefore, immune checkpoints in regulating T cell activation and immune tolerance have been widely studied. More recently, a new frontier in anticancer [6, 19–21] and antiviral therapy [22] has emerged, in which these receptors are being targeted to improve T cell responses [23–25].

### CTLA-4 as a coinhibitory receptor for T cell activation

The process of T cell activation is tightly regulated by costimulatory signals for full activation, and it is also regulated by coinhibitory signals [26]. The main costimulatory signals for T cell activation are from the B7-1 or B7-2 molecules on antigen presenting cells, which can bind to CD28 on T cells. After binding to its specific antigen ligand, the resulting TCR signals in conjunction with the costimulatory signals from CD28/B7 interaction lead to fully activation of T cells and production of cytokines [27]. CTLA-4 is a major coinhibitory receptor in regulation of T cell response during the priming phase [28]. In contrast to CD28, CTLA-4 delivers an inhibitory signal, and it has a much higher affinity for B7 than CD28 [29, 30]. Thus, CTLA-4 competes for binding to B7, and thereby prevents CD28-mediated T cell costimulation, and also inhibits T cell activation [29, 31, 32]. Moreover, CTLA-4 can capture B7, which induces the degradation of these ligands within the cell via trans-endocytosis [33]. All these effects dampen T cell activation and enhance immune tolerance. In addition, CTLA-4 is essential for the regulatory T cells (Tregs) function [34, 35]. Tregs require CTLA-4 to maintain their function to suppress immune responses, and deficiency of CTLA-4 results in development of profound systemic autoimmune diseases [10, 11]. The concept of using immune checkpoint inhibitors to break T cell dysfunction in tumor patients appears to be an intriguing approach in cancer therapy. This was first demonstrated by the success of Ipilimumab, an anti-CTLA-4 mAb, resulting in the approval of Ipilimumab by the FDA for advanced melanoma [2]. All these results indicate a major conceptual breakthrough in cancer immunotherapy. Immune checkpoint blockade is game-changing and revolutionary in at least in two senses. First, the target for therapy is on immune cells but not tumor cells. Second, the approach is not to attack tumor-specific antigens but to remove an inhibitory pathway.

### PD-1 plays a key role in inhibiting the effector function of antigen-specific CD8 T cells in chronic viral infections and cancer

In chronic viral infections and cancer, T lymphocytes are under persistent exposure to antigen stimulation. This is commonly associated with progressive deterioration of T cell effector function with constitutive coinhibitory receptor expression by T lymphocytes, a state called ‘exhaustion’. It usually manifests as a gradual loss of effector functions and cytokine production, as well as persistently increased expression of multiple inhibitory receptors [36–38]. T cell exhaustion was demonstrated in chronic viral infections such as human immunodeficiency virus (HIV), hepatitis C virus (HCV), and hepatitis B virus (HBV), and in cancer conditions [36, 38–41]. Exhausted T cells are characterized by deficits in proliferation and in the activation of effector functions (cytotoxicity and cytokine production) upon antigen stimulation [42]. Coinhibitory receptors are highly expressed on dysfunctional or exhausted T cells. The inhibitory ligands that regulate T cell function and induce T cell exhaustion/dysfunction in tissues usually exhibit increased expression on cancer cells and virus-infected cells within tissue microenvironments.

The immune checkpoint molecule PD-1 was originally identified from a T-cell line as a novel member of the immunoglobulin gene superfamily with an immunologic receptor tyrosine-based inhibitory motif (ITIM) [43]. Initially, PD-1 was demonstrated to be a receptor for cell death; however, the PD-1 pathway was later found to play a regulatory role in inhibiting T cell activation and restraining T cell function [12, 13]. Accumulating evidence indicates that the PD-1 pathway is critical in inhibiting viral antigen-specific CD8 T cells in chronic HIV [44], HCV [45], and HBV infections [25, 46]. Recent studies demonstrated that the interaction between PD-1 on T cells and its ligands plays an important role in inducing T cell exhaustion and dysfunction. Restoration of T cell function by PD-1 blockade supported the importance of this inhibitory pathway in animal models of viral infection [25, 41, 47, 48]. Moreover, it was shown that targeting PD-1 and other immune checkpoints is able to reverse this dysfunctional state and reinvigorate T cell activity in chronic viral infections and cancer [6, 24, 36, 38, 41, 49, 50].

### Multiple inhibitory receptors are expressed by “exhausted” T cells in cancer and chronic viral infections

Whereas inhibitory receptors can be transiently expressed by effector T cells during the activation stage; persistent overexpression of inhibitory receptors is a hallmark of exhausted T cells [51–54]. So far, the molecular mechanisms by which inhibitory receptors regulate T cell exhaustion are still unclear. In addition to PD-1, exhausted T cells also express multiple inhibitory receptor molecules on their cell surface [42]. These inhibitory receptors include the lymphocyte activation gene 3 (LAG-3) protein, T cell immunoglobulin- and mucin-containing molecule-3 (Tim-3), CTLA4, and many other inhibitory receptors [49]. In fact, a core set of inhibitory receptors, including PD-1, LAG-3,Tim-3, and the T cell immunoglobulin and ITIM domain (TIGIT, also known as Vstm3 and WUCAM), is also expressed on tumor-infiltrating lymphocytes (TILs). Other combinations of inhibitory receptors, such as PD-1 and Tim-3 [55, 56] are also co-expressed in exhausted/dysfunctional T cells to regulate their function. Taken together, the accumulating results on these inhibitory receptors in co-regulation of T cell dysfunction suggest that these coinhibitory pathways may play different roles in T cell exhaustion.

Recent genomic studies exploring the transcriptional profile underlying T cell exhaustion revealed that exhausted T cells have a transcriptional profile with major alterations in the expression of inhibitory receptors, cytokine and chemokine receptors, signaling molecules, transcription factors, and genes involved in T cell metabolism [37, 57, 58]. Although considerable advances in mechanistic study have been made in the past few years, the molecular mechanisms of T cell dysfunction/exhaustion are still not clear. In addition, there still lacks a clear understanding of the intriguing molecular pathways involved in the reversal of T cell exhaustion/dysfunction. In fact, we have only just begun to understand the transcriptional coordination of T cell exhaustion. Moreover, accumulating studies have emphasized the pivotal importance of T cell metabolism in regulating T cell dysfunction/exhaustion [59–61]. This has prompted intense exploration into the targeting of other immune checkpoints or coinhibitory receptors besides PD-1 and CTLA4. Among them, LAG-3, Tim-3, and TIGIT are emerging immune checkpoints under preclinical and clinical development for cancer therapy.

### LAG-3

Among the new immune checkpoints, LAG-3 was originally cloned in 1990 as a membrane protein upregulated on activated T lymphocytes, and natural killer (NK) cells [62]. LAG-3 gene has high homology with CD4 and structurally resembles the CD4 molecule. Similarly, LAG-3 binds to MHC class II with a higher affinity [63]. In addition to MHC class II, LSECtin, a DC-SIGN family molecule, was suggested to be another ligand for LAG-3 [64]. The most well known feature and function of LAG-3 are its role in the negative regulation of T cell response, and this makes it a potential target for immune modulation. LAG-3 is highly expressed on both activated natural regulatory T cells (nTreg) and induced FoxP3^+^ Treg (iTreg) cells [65]. Blockade of LAG-3 abolishes the suppressor function of Treg cells. Moreover, LAG-3 is crucial for Treg cell-mediated T cell homeostasis [66, 67]. All these results support a functional role for LAG-3 in Treg cell function. In cancer and chronic viral infections, expression of LAG-3 is increased in exhausted T cells [49]. PD-1 and LAG-3 are co-expressed on dysfunctional or exhausted virus-specific CD8^+^ T cells [68], and on both CD4^+^ and CD8^+^ tumor infiltrating lymphocytes (TILs) in animal models of cancer [69]. Blockade of LAG-3 can enhance anti-tumor T cell responses [70]. Co-blockade of the LAG-3 and PD-1 pathways is more effective for anti-tumor immunity than blocking either molecule alone [69, 71]. Therefore, in both chronic viral infections and cancer, PD-1 and LAG-3 signaling pathways functionally cooperate to inhibit T lymphocytes responses. The potential for LAG-3-driven immuno-modulatory responses are currently being explored for clinical cancer therapy.

### Tim-3

Tim-3, another newly defined immune checkpoint, was first identified as a T cell surface molecule expressed selectively in interferon (IFN)-γ-producing T cells [72]. It is also expressed in innate immune cells (DCs, NK cells, and monocytes) and Treg cells [73]. Tim-3 blockade was shown to exacerbate experimental autoimmune encephalomyelitis (EAE) [72]. Studies with Tim-3 knockout mice and wild-type mice treated with Tim-3-blocking antibody demonstrated that Tim-3 signaling is required for tolerance induction and that Tim-3 blockade enhances the development of autoimmunity [74, 75]. Galectin-9, a C-type lectin, was first discovered as a Tim-3 ligand [76]. Triggering of Tim-3 by galectin-9 induced the death of Tim-3^+^ T cells and reduced EAE disease severity [76]. Most recently, CEACAM-1 was also identified as a novel cell surface ligand for Tim-3 [77]. CEACAM-1 co-immunoprecipitates with Tim-3, and it is co-expressed with Tim-3 on CD8^+^ TILs that exhibit the dysfunctional/exhausted phenotype. The regulatory function of Tim-3 is abrogated in the absence of CEACAM-1, suggesting a requirement of CEACAM-1/Tim-3 co-expression and interaction for optimal Tim-3 function [77].

The interleukin (IL)-27/NFIL3 axis was identified as a crucial regulator of effector function of T lymphocytes via induction of Tim-3 and the immunosuppressive cytokine IL-10 [78]. Tim-3's function in T cell exhaustion was recently examined in both chronic viral infections and cancer. The observation that Tim-3^+^ CD8^+^ T cells exhibit the dysfunctional/exhausted phenotype raised the question of whether PD-1 expression can be used as the sole hallmark for identifying dysfunctional/exhausted CD8^+^ T cells in chronic viral infections or cancer. In HIV infection, Tim-3 was found on dysfunctional/exhausted T cells that lacked PD-1 expression. Furthermore, Tim-3 was expressed in the most dysfunctional/exhausted population among CD8^+^PD-1^+^ T cells in several chronic viral (HCV and HBV) infections in humans and also in animal models [55, 79–81]. All these observations suggest that PD-1 and Tim-3 have non-redundant and synergistic functions in inhibiting effector T cell activity. In addition, studies on Tim-3 also indicate the presence of dysfunctional/exhausted CD8^+^ T cells in cancer. It was shown that populations of CD8^+^ TILs expressing both Tim-3 and PD-1display different functional phenotypes. Among these populations, Tim-3^+^PD-1^+^ double-positive TILs exhibit more dysfunctional or exhausted phenotypes than do Tim-3^+^ or PD-1^+^ single-positive TILs. In contrast, Tim-3-PD-1 double-negative TILs exhibit good effector function [56]. In support of these observations, co-blockade of the PD-1 and Tim-3 pathways was shown to be a more effective approach than blocking PD-1 alone for improving antitumor function and suppressing tumor progression in preclinical models of cancer. Taken together, the current data suggest that Tim-3 plays a crucial role in regulating antitumor T cell immunity [56, 82, 83].

### TIGIT

TIGIT, a recently defined new immune checkpoint, was first identified as a novel CD28 family molecule [84–87]. TIGIT is an immunoglobulin (Ig) superfamily receptor that functions as a coinhibitory receptor, and is specifically expressed by immune cells [85–87]. TIGIT is expressed by activated T cells, and is also expressed on Treg cells, memory T cells, NK cells, and follicular T helper (Tfh) cells [84–89]. TIGIT binds two ligands, CD112 (PVRL2, nectin-2) and CD155 (PVR), and these ligands are expressed by T cells, APCs, and tumor cells [84–86, 90, 91]. Genome-wide association studies have linked TIGIT to multiple human autoimmune diseases including type 1 diabetes, multiple sclerosis, and rheumatoid arthritis [92, 93]. The function of TIGIT was therefore initially investigated in autoimmunity and tolerance. In addition to its protective role in autoimmune diseases, TIGIT was also explored in cancer and chronic viral infections. The TIGIT ligands, CD112 and CD155, are expressed in many tumor cells. In addition, the positive counterpart of this costimulatory pathway, CD226, promotes cytotoxicity and enhances antitumor responses [94, 95]. The TIGIT-deficient mice showed significantly delayed tumor progression in different tumor models, suggesting that TIGIT negatively regulates antitumor responses [96]. TIGIT is highly expressed on TILs in the tumor microenvironment across a broad range of tumors [96–98]. TIGIT^+^CD8^+^ TILs co-express PD-1, LAG-3, and Tim-3 and exhibit the most dysfunctional phenotype among CD8^+^ TILs in murine tumors [96]. TIGIT synergizes with PD-1 and also with Tim-3 in impairing anticancer immunity [96]. Therefore, co-blockade of either TIGIT plus PD-1 or TIGIT plus Tim-3 enhances anti-cancer immunity and induces tumor regression. Taken together, these results indicate that TIGIT synergizes with other inhibitory molecules to suppress effector T cell responses and promote T cell dysfunction.

### Immune effector cells acquire inhibitory receptors in the tumor microenvironment

The ligands and inhibitory receptors that regulate T cell effector functions are mostly overexpressed on tumor-infiltrating immune cells or on tumor cells in the tumor microenvironment. Therefore, targeting these ligands and receptors is relatively specific to tumors compared to normal tissues. It is within these tumor microenvironments that immune effector cells acquire inhibitory receptors, resulting in T cell dysfunction. Soluble molecules include cytokines with immunosuppressive activity, such as IL-10, transforming growth factor (TGF)-β, and IL-27, which regulate immune responses to tumor cells and induce T cell dysfunction within the tumor microenvironment [99–102]. The IL-10 pathway has been intensively studied for its role in T cell dysfunction in chronic viral infections and cancer [99, 100]. IL‑10 promotes T cell exhaustion, and IL‑10 blockade reverses T cell dysfunction during chronic viral infections [99]. Co-blockade of both IL-10 and the PD1 reverses CD8+ T cell exhaustion and enhances viral clearance, which supports a role for IL-10 in T cell exhaustion [101]. Moreover, inhibition of TGFβ signalling in CD8+ T cells in vitro restores the dysfunction of exhausted T cells [103]. However, systemic blockage of TGFβ by treatment with its inhibitor or blocking antibody had only little benefit [104]. The type I IFNs (IFNα/β) are crucial in innate antiviral effects; however, recent reports demonstrated that type I IFNs signaling paradoxically facilitated viral persistence by enhancing immune suppression during chronic infection, and IFNα/β blockade reversed T cell exhaustion in chronic viral infection [105, 106], All these data suggest a possible role for IFNα/β in promoting exhaustion. Thus, chronic exposure to IFNα/β enhances T cell exhaustion/dysfunction during chronic infections. In recent studies, it has been demonstrated that the immunosuppressive cytokine, IL-27, is a potent inducer of Tim-3^+^ exhausted/dysfunctional T cells and a promoter of tumor growth in mice model [78]. Furthermore, IL-27 signaling directly controls expression of Tim-3 via induction of NFIL3, a transcription factor, which is critical for the development of exhausted/dysfunctional T cell phenotype [78]. Moreover, IL-27 is an inducer of the “coinhibitory” gene module in effector T cells. IL-27 induces inhibitory molecules including PD-1, Tim-3, LAG-3, TIGIT, and IL-10, which overlap with the mediators of T cell exhaustion, in chronic viral infections and cancer [101, 102]. From these observations, IL-27 signaling pathway may regulate the suppression program that drives the development of T cell exhaustion in cancer and chronic viral infections.

Besides inhibitory receptors on the cell surface, there are other soluble immune-inhibitory molecules within the tumor microenvironment. These soluble immune inhibitory molecules include certain metabolic enzymes, such as arginase produced by myeloid-derived suppressor cells (MDSCs), and indoleamine 2,3‑dioxygenase (IDO), which are expressed by both cancer cells and tumor-infiltrating myeloid cells [107–110]. Moreover, FOXP3^+^ CD4^+^ Treg cells also influence effector T cell function in the microenvironment within tumor. However, exactly how Treg cells affect the induction of T cell dysfunction has not been well defined. Besides FOXP3^+^CD4^+^ Treg cells, other immune cell types, such as NK cells, immunoregulatory APCs, MDSCs [111, 112], and CD8^+^ regulatory cells [113, 114], may affect tumor progression and directly or indirectly enhance T cell dysfunction.

CTLA4 and PD-1, the two immune checkpoint targets that have been extensively studied in clinical immuno-oncology, regulate anticancer T cell responses via different mechanisms and at different levels. This implies that anticancer immunity can be enhanced at multiple levels and by different mechanisms. It also implies that combination strategies for cancer immunotherapy can be wisely designed based on mechanisms and on results obtained from preclinical models. A better understanding of the specialized regulatory roles of these receptors and definition of the mechanisms of T cell dysfunction will provide more insights for rational design and development of cancer immunotherapy that target these receptors.

## Conclusion

Recent studies demonstrated that immune checkpoint inhibitors are able to induce durable, long-lasting cancer control. These antibodies have moved immuno-oncology therapy into a new era and indicate that modulation of immune response is a crucial therapeutic strategy for cancer treatment. Although current immunotherapies targeted at the immune checkpoints, PD-1 and CTLA-4, exhibit enormous potential to control cancer, there are still some tumor types and many patients that remain largely refractory to these therapies. This has prompted intense investigation into the targeting of other immune checkpoints or coinhibitory receptors in order to increase the therapeutic repertoire. A diverse array of T cell co-receptors are now being explored for developing new potential targets for clinical cancer therapies. There are multiple additional immune checkpoints which represent new potential targets for cancer immunotherapy, and they are now under active development. These include antibodies targeting new immune checkpoints, particularly LAG-3, Tim-3, and TIGIT [73]. They also include agonist antibodies against activating receptors such as CD137, CD27, ICOS, GITR, B7-H3, and others. Rational combinations of immune checkpoint inhibitors with other immunotherapies are also tested in ongoing studies [19]. In addition, new biomarkers that help to select patients for particular types of immune checkpoint therapy are under intense investigations. A clearer understanding of the regulatory roles of these immune checkpoints and elucidation of the mechanisms of T cell dysfunction will shed insights on the development of new therapies for cancer treatment.

## Abbreviations

APC: Antigen presenting cells
CTLA-4: Cytotoxic T lymphocytes antigen- 4
DC: Dendritic cell
EAE: Experimental autoimmune encephalomyelitis
HBV: Hepatitis B virus
HCV: Hepatitis C virus
HIV: Human immunodeficiency virus
IFN: Interferon
LAG-3: Lymphocyte activation gene 3 protein
MDSC: Myeloid-derived suppressor cells
MHC: Major histocompatibility complex
NK cell: Natural killer cell
PD-1: Programmed death-1
TCR: T-cell receptor
TIGIT: T cell immunoglobulin and ITIM domain
TIL: Tumor infiltrating lymphocyte
Tim-3: T cell immunoglobulin- and mucin-containing molecule-3
Treg: Regulatory T cells
